# Refitting Temporary Plates

**Published:** 1861-03

**Authors:** J. Richardson


### REFITTING TEMPORARY PLATES.

BY J. RICHARDSON.

Inasmuch as the utility of temporary sets of artificial teeth is so generally recognized by practitioners everywhere, and as most patients desire them, and as the usefulness of these appliances is commonly very much impaired by the changes which take place in the form of the alveolar ridges during the progress of absorption, it would seem desirable that some available means should be devised to re-adapt them to the parts without the labor and inconvenience of remodeling the entire piece. While most patients, in a full knowledge of the disabilities oftentimes incident to the use of temporary substitutes, request to be supplied with them, yet there is not an inconsiderable class, skeptical of their utility for the reason mentioned, and disinclined to submit to the annoyance of an ill-fitting plate, who prefer to forego the use of artificial teeth during the entire period of absorption.

To increase the usefulness of these pieces and render them more desirable, either of the following methods may be adopted to re-fit, from time to time, with the greatest certainty and facility, such as have lost, in a greater or less degree, their adaptation to the parts in the mouth.

*First method.*—Take, for example, a full upper set on either gold, silver, or vulcanite. Secure, in the first place, an accurate impression of the mouth in its changed condition in plaster, and from this a plaster model in the manner usually practiced. Perforate the palatal portion of the plate with from eight to twelve holes at different points, and also the external borders, from heel to heel of the plate, at intervals of from one-eighth to half an inch apart, and near the edges. These holes may be enlarged to the dimensions of a medium sized knitting-needle; or if the piece is of vulcanite, to twice or three times that size. On the lingual and buccal surfaces the holes are well counter-sunk with a bur drill. The plaster model, with the central portion raised to form a chamber (and which should be made to correspond, as nearly as possible, in position, form and thickness, with the chamber in the plate, if one exists), is next heated throughout by placing it over a spirit flame, or in the baking furnace of an ordinary cooking stove, or the muffle of a furnace, and when of a temperature that will barely admit of being taken in the hand, remove and cover the face of it with a sheet of India rubber or gutta percha as prepared for vulcanite work, and press it down upon the face of the model with the fingers. Apply the perforated plate to the model, being careful to secure a proper relation of the two; then press the former down firmly upon the model. To render the vulcanite material still more plastic and compressible, the whole may now be returned to the furnace, and subjected to a uniform heat throughout, when it may be removed and firm and steady pressure made upon the plate and teeth until forced as nearly as practicable into contact with the face of the model. Portions of gum will be forced through the apertures and out at the borders of the plate; these should be well packed into the countersinks and under the edges of the plate, when the model, with the rubber and plate adherent, may be placed in a vulcanizing flask and encased bodily in plaster. It is then placed in a heater and vulcanized. If all the steps in the process have been carefully conducted, the fit of the plate will be perfectly restored, with no material change in the antagonism, or none, at least, that is not susceptible of ready correction. The union between the vulcanite lining and the plate will be strong and lasting, and altogether impermeable to the fluids of the mouth.

In the case of lower pieces, the holes should be made along the external and internal borders of the plate near the margins. In all other respects, the manipulations are the same as those described above.

It is scarcely necessary to observe that, in the use of gold plates, the method is inapplicable whenever it is designed to re-swage the same plate for the permanent piece.

*Second method.*—Perforate the plate whether of gold, silver, or vulcanite, as before directed, and employing this as a cup or holder, take an impression of the mouth in plaster, pressing the plate up closely to the parts. The plaster forced through the holes and filling the countersinks on the opposite side of the plate, will serve to bind the plaster to the plate and prevent, with cautious manipulation, the two from separating as they are being detached from the mouth. When removed, the plaster impression lining the plate is trimmed even with the borders of the latter and then varnished and oiled. The lower section of a vulcanizing flask is now filled with a batter of plaster on a level with its upper surface, and the impression, filled with the same, is turned over and placed in the center of the flask, with the edges of the plate touching the surface of the plaster. The plate and adhering plaster are now carefully separated from the model. After cutting out the plaster from the holes and countersinks in the plate, the plaster forming the impression is detached from the plate, and the holes and countersinks filled with wax. The plate is then re-adjusted over the model, and (the surrounding surface of the plaster in the flask having been varnished and oiled), plaster is poured in upon the upper surface of the plate and teeth, filling the upper ring. When the plaster is sufficiently hard, the two sections of the flask are separated, and grooves formed running out from the matrix to the margins of the flask. A sufficient quantity of vulcanizable rubber is now either placed upon the model or packed in upon the palatal surface of the plate,—before doing which, however, the wax filling the holes and counter-sinks in the plate, (and which was placed there to prevent portions of plaster last poured in forming the matrix from running in and filling them up,) should be worked out with a small instrument. The whole being sufficiently heated, the two sections of the flask are forced together, expelling redundant material. The piece is then vulcanized as in the former case.

The above method, though somewhat more complicated than the former, is quite simple in its details, and will occupy but little more time, and is, withal, more certain in its results. For the idea of forming a matrix substantially as described, I am indebted to Mr. R. A. Mollyneaux, an intelligent member of the present class of the Ohio Dental College.

